# Appendix

**Published:** 1826-06

**Authors:** 


### APPENDIX.

We have received the following from Dr. Venables, relative to the Review of his Work on "Diabetes." We publish it, that he may have no reason to complain of injustice; while we give it as an Appendix, that it may not interfere with the usual arrangement of the Journal. (Editors.)

In the Review of my work on Diabetes, which has appeared in the London Medical and Physical Journal for the present month, an opinion of its merits seems to have been formed upon very erroneous assumptions. The Reviewer first finds fault with me because I did not choose to communicate to the profession my views with regard to diabetes through some other medium than that of a distinct and specific publication. Upon this question, however, it is possible that the ideas of the Reviewer might prove as injudicious as the course which I have adopted. It is asserted, that two simple facts are issued forth in the cumbersome and bulky form of a large octavo volume. The body of the work consists of ninety-four pages; and in these ninety-four pages I have enumerated causes, and circumstances in the general history of the disease, which I cannot find enumerated, or even adverted to, in systematic works, nor in the most recent publications upon the subject; but yet systematic works generally profess to give a summary of every thing known upon each specific subject. It is unnecessary to enumerate these individually, as, on a reference to the proper heads, they will be immediately recognised.

I have stated that an increased flow of urine is frequently a severe disease in children, and that this circumstance has not attracted that general attention which its importance demands. How does the Reviewer attempt to prove the falsehood of the latter proposition? By asserting that Dr. Underwood has devoted a chapter to Polydipsia, and another to Diabetes infantilis; that he quotes Zuingerus, as testifying that thirst, accompanied with a great flow of urine, is a common affection among children; and that Moreton, in his Phthisiologia, mentions consumption from diabetes as a common disorder in infancy. These facts, in my mind, only tend to confirm the truth of my assertions, and to establish the accuracy of my observations as to the frequent prevalence of such an affection; for the authors above referred to clearly prove the existence of such a disease, and distinctly state (according to the Reviewer,) that "it is a common affection among children." So far for the prevalence of the disease: now for the professional attention bestowed upon it.

The Reviewer has not referred specifically to any cases recorded in the public journals, or other works, to show that the disease, as I have described it, has met with that universal attention which the Reviewer would lead us to suppose, or that it has even met with that ordinary attention bestowed upon diseases attended with more obvious symptoms. The Reviewer states that he has enjoyed extensive opportunities of witnessing the diseases of early life: surely, then, a reference to such experience, and to the journals of the public institutions, would have afforded the most convincing proofs of the accuracy or inaccuracy of my statements as to the professional attention which the disease has received. I would almost venture to abide by such reference: I mean a reference to the admissionbooks of the public institutions. But even if, upon such investigation, it should appear that cases of the disease as described have been noted, still I should be justified in relating my cases, because, although the cases which might have been admitted to these establishments had been noted in their records, yet the particulars had not been submitted to the public.

It is objected that I have related only eleven cases, the date of the earliest being 1818, and that I have no where stated that I have seen more. Now, eleven well-authenticated cases, the nature of which has been clearly and distinctly defined, (and surely I have taken sufficient pains to establish the nature or nosological character of these cases,) are as sufficient to prove the existence of a disease, and to estimate the efficacy of any remedy, as eleven hundred, or even eleven thousand. Surely the Reviewer must have perceived that the selection of cases was made with the view not only of proving the efficacy of the phosphate of iron, but also of illustrating the action of the causes which I have enumerated as capable of exciting the disease. It is rather singular to find the Reviewer decidedly differing as to the causes which I have enumerated, and yet declaring that my views are nothing more than a transcript of those of preceding observers. Of the efficacy of the phosphate of iron in reducing the flow of urine, even in the most confirmed diabetes, I have lately had a most satisfactory and convincing proof. The case occurred in the practice of my friend, Mr. Jeston. It is that of a married woman, whose mother died of this disease, and in whose family it seems to have prevailed as an hereditary affection. The quantity of urine discharged in twenty-four hours amounted to between five and six, and sometimes seven quarts. It was so sweet as to resemble syrup, and the specific gravity amounted to 1.047, and sometimes 1.049. The phosphate of iron has repeatedly reduced the quantity of urine from twelve to five pints,* and even under. The specific gravity also was considerably reduced, and at one period the urine had lost its sweet taste.

The Reviewer seems to run into an extreme the very opposite of that which he condemns in me. He accuses me (though I think very unjustly) of looking upon every disease with which diabetes may happen to be either complicated or co-existent, as secondary to it; and criticises me for precipitancy in excluding such antecedents as pulmonic diseases from the class of what are termed, in medical language, "causes." With respect to those diseases which may happen to prevail at the same time with diabetes, they may be either complicated with it,—that is, so related as to be either causes or consequences,—or they may be merely coexisting affections, wholly independent of each other, and in no way related as cause and effect. Before the Reviewer censured me, he should, either from his own experience or that of others, have established, first, the relation between diabetes and those co-existing affections; and, secondly, that the co-existing affections are actually and truly antecedent diseases. It is consistent with my own experience, and I believe it will be confirmed by a reference to the clearest and best histories of the disease, that diabetes may have existed for a long time without any remarkably severe derangement of the health; and even the fever, thirst, and voracious appetite, are frequently absent, or prevail to such a trifling extent as to excite but little attention. But still further, I believe it will be allowed that the diseases of other organs seldom appeared till the health had been considerably undermined by the disease of the kidneys. In the morbid anatomy of the disease, it is well established that the kidneys are invariably diseased to a greater or less degree, but still always severely. If we pursue the inquiry further, we shall find that other organs are frequently involved in disease, but there is no precise order,

* Opium was also freely administered.

either of parts or of morbid condition; so that in some instances one organ is affected, in other cases another; in some the morbid condition is of one kind, in others of a different. Upon this subject I must beg leave to quote a passage from the last edition of Dr. Hooper's Dictionary:—"The liver, pancreas, spleen, and stomach are, *in general*, perceived to be in a natural state: when they are not so, the occurrence is considered to be accidental." How far this passage corroborates my views, I shall leave the reader to decide.

It is stated that gin comes under my ban. It is a singular circumstance, (of which, however, I was not aware till the work had been published,) and one which tends strongly to confirm the propriety of the denunciation, that English gin is highly adulterated with turpentine. That such a preparation is calculated to excite the kidneys, the Reviewer, I have no doubt, will willingly concede. In a conversation some little time since with my friend Mr. Brooks, he mentioned that bricklayer's labourers were more subject to diseased kidneys than the generality of other persons.* Bricklayer's labourers are the persons who prepare the mortar, or work up the caustic lime into this cement, and are therefore exposed to the alkaline effects of the lime in the most eminent degree. When handed to the bricklayer as mortar, these properties are nearly neutralised. We know that the individuals engaged in smelting and working the metallic ores containing arsenic and lead, are extremely liable to paralysis, colic, and the other diseases which arise from the internal use of these poisonous minerals. May not the peculiarity of disease to which bricklayer's labourers are subject, be accounted for upon similar principles. This observation I have adduced as strongly corroborating my views with respect to the deleterious influence of the alkalies upon the kidneys.

I have not been so fortunate as to have had an opportunity of consulting Dr. Latham's work, and therefore, whatever coincidence of principle or experience may exist, must be highly gratifying to me. Had I, indeed, been constructing a SYSTEMATIC TREATISE upon Diabetes, I should have felt it incumbent on me to consult every authority upon the subject; but I have distinctly stated that I send it forth as a mere practical summary, furnished entirely from the resources of my own experience. The phosphate of iron is not mentioned as a remedy for diabetes in any work, systematic or specific, upon this disease, to which I have had access; nor is it mentioned, or even its use adverted to, by Prout, the latest writer upon this disease. Surely, if Dr. Latham's suggestion had been more generally known, the different writers upon the disease would have taken some notice of the remedy; and, as my experience is so ably supported by Dr. Latham's, the publication of my cases may tend to bring a neglected but really efficient remedy into more general notice, and thus extend its use. So far allowing that Dr. Latham's application of the remedy and mine were precisely the same. But I maintain, and I rest my assertion upon the passage selected by the Reviewer himself, (not having seen the Doctor's work,) that my application of the phosphate differs, *toto cœlo*, from that of Dr. Latham. "For the purpose of *restoring the vigour of the system, when the diabetic tendency has been checked, nothing would perhaps be better* than iron combined with phosphoric acid, as it is found in the phosphate and oxyphosphate of that powerful mineral; and, when a purgative is wanted, soda phosphorata should always be preferred. In chronic diabetes, more especially, I have seen this plan very beneficially employed." From this passage, therefore, it appears that Dr. Latham merely suggests its use as a tonic when diabetes

---

* Mr. Brooks has been thirty years in practice, and the observations of thirty years' professional experience are entitled to every respect.

has been cured. I recommend its use from the very beginning, as forming a powerful part of the means for effecting that cure; and the cases which I have related fully warrant that recommendation, and may prove further useful by exciting more general attention to Dr. Latham's work. But should it even appear that Dr. Latham has anticipated me upon this subject, so far from feeling either chagrin or disappointment, I should be truly rejoiced to find myself so ably supported.

The last observation I shall make upon this subject is the following:—" Another plan of treating the disease, (says Dr. Hooper, in the last edition of his Dictionary,) has been more recently proposed, namely, by bleeding and other antiphlogistic means, and some cases of its success have been recorded; BUT FURTHER EXPERIENCE IS CERTAINLY REQUIRED, before we should be justified in relying much upon it." Surely, where there exists such indecision and doubt upon the propriety of antiphlogistic means in the treatment of this disease, the history of eighteen cases, in which antiphlogistic means formed so prominent a part of the method of cure, must have some interest.

Evidently the Reviewer thinks very meanly of this production: however, it is consoling to me to reflect, that I hold in my possession the opinion of professional authority, I have no hesitation in asserting, as respectable as the Reviewer, and which has pronounced far more favourably of its merits. Notwithstanding, I have no objection " to drink at the fountain-heads," while Latham and Prout supply the stream.

*Henley-upon-Thames;*

*10th March*, 1826.

